# Single-cell RNA sequencing analysis reveals alginate oligosaccharides preventing chemotherapy-induced mucositis

**DOI:** 10.1038/s41385-019-0248-z

**Published:** 2020-01-03

**Authors:** Yong Zhao, Yanni Feng, Ming Liu, Liang Chen, Qingshi Meng, Xiangfang Tang, Shukun Wang, Lei Liu, Lan Li, Wei Shen, Hongfu Zhang

**Affiliations:** 1 State Key Laboratory of Animal Nutrition, Institute of Animal Sciences, Chinese Academy of Agricultural Sciences, Beijing, 100193 P. R. China; 2 College of Life Sciences, Qingdao Agricultural University, Qingdao, 266109 P. R. China; 3 College of Veterinary Sciences, Qingdao Agricultural University, Qingdao, 266109 P. R. China

## Abstract

Worldwide the incidence of cancer has been continuing increasing. Mucositis of the gastrointestinal tract is a common side effect in patients under chemotherapy. Anticancer drug busulfan, used for treating chronic myeloid leukemia especially in pediatric patients, causes mucositis of the gastrointestinal tract. Alginate oligosaccharides (AOS) are natural products with attractive pharmaceutical potentials. We aimed to investigate, at the single-cell level, AOS preventing small intestine mucositis induced by busulfan. We found that busulfan disturbed the endoplasmic reticulum and mitochondria of cells in the small intestine, damaged cell membranes especially cell junctions, and disrupted microvilli; all of which were rescued by AOS. Single-cell RNA sequencing analysis and functional enrichment analysis showed that AOS could recover small intestinal function. Deep analysis found that AOS improved the expression of transcriptional factors which explained AOS regulating gene expression to improve small intestine function. Further investigation in IPEC-J2 cells found that AOS acts its function through mannose receptor signaling pathway. Moreover, the improved blood metabolome confirmed small intestinal function was recovered by AOS. As a natural product with many advantages, AOS could be developed to assist in the recovery of intestinal functions in patients undergoing anticancer chemotherapy or other treatments.

## Introduction

The incidence of cancer has been continuing increasing worldwide.^1–4^ Many investigations have reported that mucositis of the gastrointestinal (GI) tract is a common side effect and occurs in ~40% of cancer patients under chemotherapy.^1–4^ Intestinal mucositis is characterized by decreased villi length, and disruption of crypt cell homeostasis and tight junction proteins in the small intestinal mucosa.^2,4^ The epithelium of the mammalian small intestine is a highly ordered and structured tissue with repeated crypt-villus units along the axis. Intestinal stem cells are located at or near the base of crypts and divide to produce transit-amplifying cells (TAs). TAs then develop, following proliferation and differentiation, into five main cell types (enterocytes, goblet cells, Paneth cells, enteroendocrine (EED) cells, and tuft cells).^5–8^ Enterocytes, the most numerous villus cell type, produce the digestive enzymes and transporters for the digestion and absorption of nutrients, respectively, and also protect the body from the harsh bacterial-rich environment.^5,9,10^ Goblet cells and Paneth cells play very important roles in mucosal defense because they are mucus-secreting cells and defensin-secreting cells, respectively. EED cells regulate hormone secretion to control GI processes. Tuft cells are chemosensory cells expressing taste receptors like α-gustducin and TRPM5.^11^ All these five types of cells are tightly structured in the crypt-villus.^5,6,11^ Mucositis may lead to morbidity and even mortality, because the GI tract is a barrier that protects the body from pathogenic microbes,^6,9,10,12–14^ and it plays vital roles in the digestion and absorption of nutrients, the secretion of mucus and hormones, and interaction with commensal microbiota.^6,10^

Alginate oligosaccharides (AOS) are excellent natural products derived from the degradation of alginate. They are attracting great attention from a pharmaceutical perspective^15–17^ because of their following benefits: anti-inflammatory,^16^ anti-apoptosis,^18^ anti-proliferation,^19^ antioxidant activities,^15,18,20^ and even anti-cancer properties.^21^ AOS benefits intestinal morphology and barrier function by increasing the length of intestinal villi, the content of secretory immunoglobulin A, and the number of Goblet cells.^22^ However, the underlying mechanisms of how AOS improve small intestine morphology and function from the single intestinal cell level is unknown.

Busulfan, an alkylating agent and an effective chemotherapeutic drug, has been used for patients with chronic myeloid leukemia especially for children (under 3 years of age). Moreover, it has been used for myeloablative-conditioning regimens before stem cell transplantation.^12,13,23^ Busulfan was used to produce the small intestine mucositis animal model in current investigation because it causes mucositis in patients.^12–14^ Many investigations have attempted to reduce chemotherapy-induced intestinal disruption by using prebiotics, probiotics, selenium, volatile oils, and others,^1,2,24,25^ however, these efforts have not been successful.^26,27^ Therefore, new approaches or new medicines are urgently needed to assist in the recovery following mucositis in cancer patients (especially pediatrics) under chemotherapy. The purpose of this investigative was to explore the improvement of small intestine by AOS after busulfan treatment and the underlying mechanisms at the single-cell level.

## Results

### AOS rescued the cellular damage caused by busulfan

**Fig. 1:**
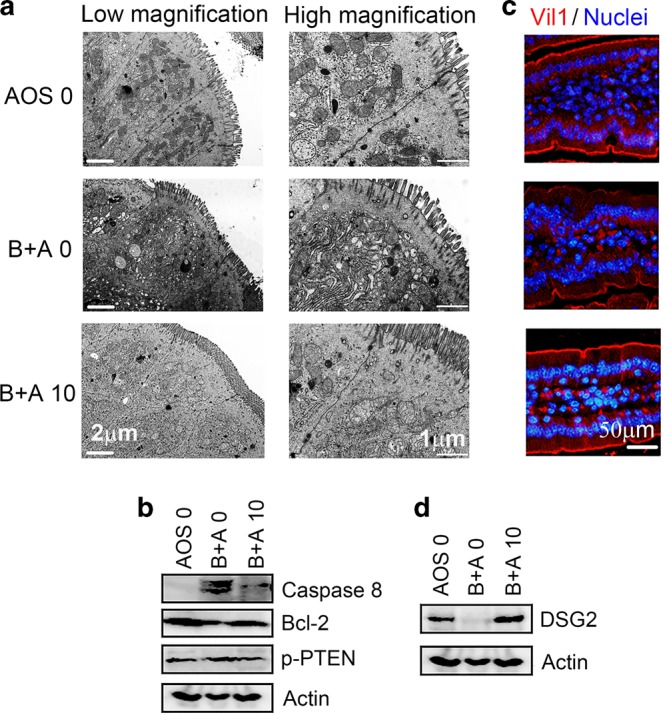
AOS rescue the cellular damage caused by busulfan. **a** TEM analysis shows AOS rescue small intestine cellular damage cell junction disruption caused by busulfan (scale bars, 1 μm for low magnification images; 2 μm for high magnification images). **b** WB analysis of protein levels of Caspase 8, Bcl-2, p-PTEN in mouse small intestine samples. **c** IHF analysis of Vil1 in mouse small intestine samples. **d** WB analysis of protein level of DSG2 in mouse small intestine samples. (*n* > 6/group).

There were four treatment groups (AOS 0, AOS 10, B + A 0, B + A 10 mg/kg body weight) in this investigation as stated in the “Materials and methods” section. AOS 10 mg/kg had some effects on the small intestine at the histopathological and ultrastructural levels, and gene expression levels. However, the beneficial effects on murine intestine was not so obvious as in the mice treated by busulfan (B + A 10 mg/kg). In order to show the rescue effects of AOS, AOS 10 mg/kg was removed from the following data analysis. From the ultrastructure of the small intestine, it was clear that busulfan treatment damaged the small intestinal cells, causing swelling of the ER and mitochondria, a decrease in the number of desmosomes on the cell membrane (cell–cell junctions), and a reduction in the density of microvilli (Fig. 1a). AOS (B + A 10) rescued the busulfan induced damage by reducing swelling in the ER and mitochondria, assisting recovery in the number of desmosomes on cell–cell junctions, and elevating the density of microvilli (Fig. 1a). Histopathology of small intestinal tissues are presented in Fig. S1a. The cellular damage caused by busulfan was also shown by an increase in the protein levels of caspase 8 and p-PTNE, with a concurrent decrease in the protein levels of Bcl-2 (Fig. 1b). However, the protein levels of caspase 8 and p-PTEN were decreased while the protein level of Bcl-2 was increased by AOS in B + A 10 (Fig. 1b). The microvilli also were recovered under AOS treatment in B + A 10 with an increase in the protein level of villi (Fig. 1c). The desmosome protein DSG2 (desmoglein 2) was significantly increased by AOS in B + A 10 compared to busulfan treatment alone (B + A 0 group; Fig. 1d) which indicated that desmosome recovery was assisted by AOS. The data here suggested that busulfan caused the small intestine mucositis which was prevented by AOS. Next, we set out to explore the underlying mechanisms by which AOS rescues cellular damage caused by busulfan.

### AOS rescued the intestinal cell population as seen by scRNA-seq analysis

**Fig. 2:**
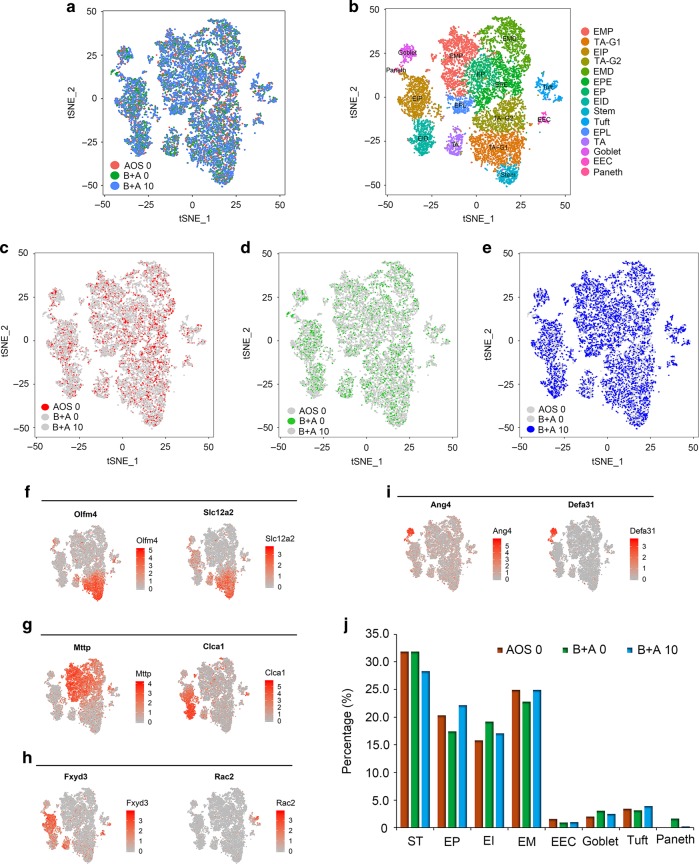
AOS rescue the intestinal cell population as seen by scRNA-seq analysis. **a** Cell distribution in scRNA-seq analysis for the cells from the three treatment groups (AOS 0, B + A 0, B + A 10). **b** Cell type clusters. tSNE of 12,653 single cells (points), colored by cluster assignment including Stem, TA (transit amplifying), TA-G1, TA-G2, EP (enterocyte progenitor), EPE (enterocyte progenitor early), EPL (enterocyte progenitor late), EIP (enterocyte immature proximal), EID (enterocyte immature distal), EMP (enterocyte mature proximal), EMD (enterocyte mature dital), EEC (enteroendocrine), Tuft, Goblet, and Paneth cells. **c** Cell distribution in scRNA-seq analysis for the cells of AOS 0. **d** Cell distribution in scRNA-seq analysis for the cells of B + A 0. **e** Cell distribution in scRNA-seq analysis for the cells of B + A 10. **f** The expression pattern of marker genes Olfm4 and Scl12a2. **g** The expression pattern of marker genes Mttp and Clca1. **h** The expression pattern of marker genes Fxym3 and Rac2. **i** The expression pattern of marker genes Ang4 and Delfa31. **j** The proportion of the fifteen clusters of cells in each sample.

The transcriptome of single intestinal cells was analyzed by scRNA-seq. After filtration, data from the three treatment groups: AOS 0, B + A 0, and B + A 10 were combined together for processing using the Seurat method (Seurat package in R Studio). The cells from these three groups were similarly distributed in the cell map (Fig. 2a, Figs. S1b–1g). The cells for individual samples are shown in Fig. 2c–e. 13,892 DEGs (different expressed genes) were detected in all the cells (Data File S1). Based on the expression of the marker genes in each cell type (Data File S1), the cells were grouped into 15 clusters as reported earlier.^28^ These 15 clusters of cells included: stem cells, transit-amplifying progenitors (TA), TA-G1, TA-G2, enterocyte progenitors (EP), enterocyte progenitor early (EPE), enterocyte progenitor late (EPL), enterocyte immature proximal (EIP), enterocyte immature distal (EID), enterocyte mature proximal (EMP), enterocyte mature distal (EMD), EEC, goblet, Paneth, and tuft cells (Fig. 2b). Gene expression of the marker genes was also specific (Fig. 2f–i, Data File S1). Olfm4 and Slc12a2 were the stem cells and TA cell markers that were highly expressed in the clusters of stem cells, TA, TA G1, and TA G2 (Fig. 2f). Mttp, as the marker gene, was highly expressed in the clusters of EM (EMD and EMP), and EI (EID and EIP; Fig. 2g). Clca1 was highly expressed in the clusters of EID and EIP (Fig. 2g). Fxyd3 was reported to be the marker gene for EEC which was also expressed in EID and EIP (Fig. 2h). Rac2 was highly expressed in tuft cells which indicated that it was a good marker gene for this type of cell. Ang4 and Defa31 are good marker genes for, and were highly expressed in, goblet and Paneth cells (Fig. 2i).

The proportion of these 15 clusters of cells in each sample was calculated using the Seurat program (Fig. 2j). Stem cells and TA cells were combined together as ST. EP, EPE, and EPL were combined together as EP. EIP and EID were together as EI. EMP and EMD were combined together as EM. The percentage of ST in group B + A 10 was lower than that in the AOS 0 or B + A 0 groups. The percentages of cells in EP and EM were decreased by B + A 0 compared to AOS 0; however, they were increased by the B + A 10 group compared to the B + A 0 group and were similar to that in the AOS 0 group. The percentage of cells in EI was increased in B + A 0 compared to AOS 0, while it was decreased in B + A 10 compared to B + A 0 and was similar to AOS 0. The percentage of EEC in B + A 0 and B + A 10 were similar, which was lower than that in AOS 0. The percentage of goblet cells was increased by B + A 0 compared to AOS 0, while it was decreased by B + A 10 to a similar level as AOS 0. The percentage of tuft cells was little changed by B + A 0 or B + A 10. However, the percentage of Paneth cells was increased dramatically by B + A 0, while it was similar in the AOS 0 and B + A 10 groups (Fig. 2j). The percentage of EEC in B + A 0 and B + A 10 were similar, which was lower than that in AOS 0 (Fig. 2j). EEC, goblet, tuft, and Paneth cells were found in much lower amounts as reported earlier.^28^ The data here indicated that AOS can improve the populations of different types of cells in the murine small intestine.

### AOS improved intestinal cell function

**Fig. 3:**
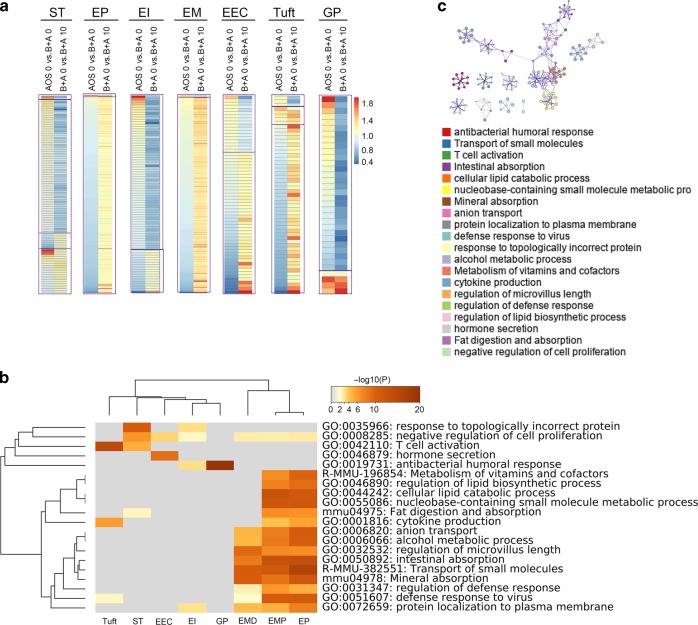
AOS improved intestinal cell function. **a** The expression pattern of the differentially expressed marker genes in each of the 15 clusters: ST (Stem, TA, TA-G1, TA-G2), EP (EP, EPE, EPL), EI EIP, EID), EM (EMP, EMD), EEC, Tuft, GP (Goblet, Paneth). **b** Multiple enrichment analysis for ST (Stem, TA, TA-G1, TA-G2), EP (EP, EPE, EPL), EI EIP, EID), EMP, EMD, EEC, Tuft, GP (Goblet, Paneth) by the online tool in Metascape. **c** Enrichment network of shared marker genes in the clusters. Each term is indicated by a circular node which are colored by cluster ID, where nodes that share the same cluster ID are typically close to each other.

The expression of these marker genes in each cluster of cells were further analyzed for their relative expression level. The expression of these marker genes in the B + A 0 group was compared to that in the AOS 0 group (AOS 0 vs. B + A 0) which was the ratio of the expression in these two groups. The expression of these marker genes in B + A 10 was compared to those in B + A 0 (B + A 0 vs. B + A 10), which gave the ratio of the expression in these two groups (Fig. 3a, Data File S1). The data showed that if the expression of the marker genes was increased (>1) in AOS 0 vs. B + A 0, the expression of the same marker genes was decreased (<1) in B + A 0 vs. B + A 10. Furthermore, it was true that if the expression of the marker genes was decreased (<1) in AOS 0 vs. B + A 0, the expression of the same marker genes was increased (>1) in B + A 0 vs. B + A 10. The data suggested that busulfan disturbed the expression of the marker genes for each cluster of cells. And busulfan increased the expression of marker genes in Paneth cells and goblet cells which further suggested that busulfan may damage these cells. On the other hand, these cells initiated the feedback mechanism to induce the defensing function of these cells. However, AOS can recover the expression of the marker genes in ST (stem cells and TA, TA G1, and TA G2 cells), EP (EP, EPE, and EPL), EI (EIP and EID), EM (EMP and EMD), EEC, tuft and GP (goblet and Paneth) cells (Fig. 3a) which indicated that AOS benefited the functions of all the types of cells in small intestine.

The differential expression marker genes from the clusters ST, EP, EI, EMD, EMP, EEC, Tuft, and GP underwent enrichment analysis (Metascape). Results showed that these marker genes were enriched into different groups specifically related to their function (Fig. 3b, c; Fig. S1i). The most enriched functional pathway for tuft cells was “T cell activation” which corresponds to the function of tuft cells.^11^ The most enriched functional pathway for EEC was “hormone secretion”. The most enriched functional pathway for GP was “antibacterial humoral response”, which is the main function of goblet and Paneth cells.^5,29^ The enriched functional pathways for ST included “response to topologically incorrect protein” and “negative regulation of cell proliferation”.^5,29^ There were 15 common enriched functional pathways for EMP and EP (EP, EPE, EPL) clusters. In addition, there were nine common enriched functional pathways for EMD, EMP and EP (EP, EPE, EPL); most of these enriched pathways are related to metabolism and absorption.^5,29^ Furthermore, the interesting enrichment was “regulation of microvillus length”, which was most enriched in EMD compared to EMP and EP (EP, EPE, EPL); this confirmed the data that microvilli were damaged by busulfan and rescued by AOS. The data in this section indicated that busulfan disrupted the specific functions of all these types of cells, however, AOS can reverse these effects correspondingly.

### Pseudo-timeline analysis revealed that AOS improved enterocyte development

Intestinal stem cells reside near the bottom of the crypt and develop into fast proliferating TA cells which then give rise to the terminally differentiated types of cells: enterocytes, EEC, goblet, Paneth, tuft cells.^5,29^ Enterocytes are the most abundant cell type in the small intestine, while all other mature cell types add up to only a few percent.^28^ Enterocytes (EP, EPE, EPL, EIP, EID, EMP, and EMD), stem cells, TA, TA G1, and TA G2 were selected for further analysis to elucidate the developmental progression of enterocytes under different treatments (Fig. S1h). Enterocyte development is asynchronous.^5,29^ therefore we aimed to examine the different stages of their development. Subsequently, we implemented the program Monocle 2 to place developing enterocytes in ‘pseudotime’’ order.^30^

**Fig. 4:**
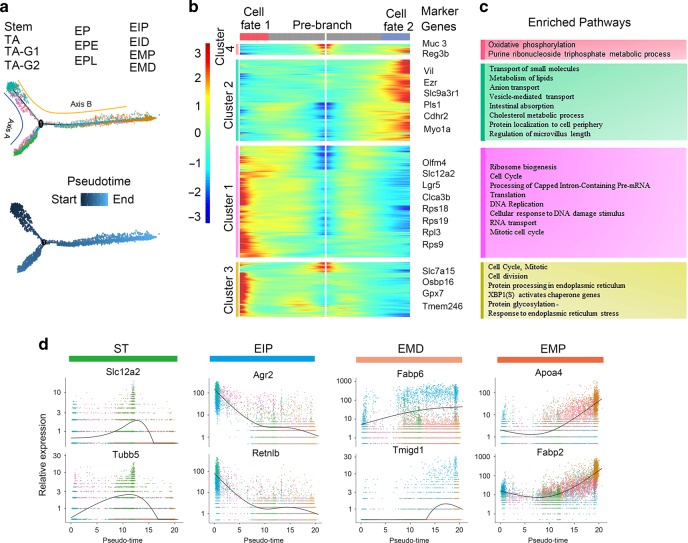
Pseudo-Timeline Analysis revealed that AOS improved enterocyte development. **a** Trajectory reconstruction of Stem, TA, TA-G1, TA-G2 and Enterocytes (EP, EPE, EPL, EIP, EID, EMP, and EMD) cells throughout chemical reprogramming discovers a bifurcation: axis A represents stem cells develop into TA (TA-G1, TA-G2); axis B shows the cells develop into enterocyte from immature to mature as the pseudo-time development. **b** Gene expression heatmap of 4292 top DEGs (cataloged in four temporal clusters) in a psudo-time order for the 11 clusters of cells (Stem, TA, TA-G1, TA-G2, EP, EPE, EPL, EIP, EID, EMP, and EMD. Representative genes are shown on the right. **c** Most enriched gene ontology (GO) terms for each of the four temporal clusters. **d** Differential expression patterns of some example marker genes from four major clusters ST, EIP, EMP, EMD.

The cells arising from stem cells were then ordered along a putative developmental trajectory from least to most differentiated enterocytes (Fig. 4a). It is noteworthy that the ordering of all these clusters of cells revealed a bifurcation of the pseudo-timeline which indicated that there were distinct molecular pathways to guide the development of these clusters of cells. In order to assess which genes regulated the progression of enterocytes along the timeline, hierarchical clustering of the genes was performed to determine the expression patterns of those that act as a function of pseudotime (Fig. 4b, c, Data File S1). Correlated with the plotted pseudo-timeline order,^30^ we found that groups of genes were expressed differentially along each axis; these genes are known to be developmentally regulated through enterocyte maturation and differentiation, including proliferating or stem cell marker genes Slc12a2 and Olfm4, and enterocyte maturation marker genes vil1 and Myo1a (Fig. 4b, c). Gene ontology (GO) analysis found that axis A was enriched for genes related to proliferation (genes in cluster 1 and cluster 3) which corresponded to the TA G1 and TA G2 clusters, while axis B was enriched for genes related to villi development and maturation (genes in cluster 2 and cluster 4) which corresponded to EP, EI, and EM clusters (Fig. 4c). The expression patterns of the marker genes for these clusters of cells are shown in Fig. 4d. The data in this section indicated that busulfan altered the cell developmental time line of the small intestinal cells which can be rescued by AOS.

**Fig. 5:**
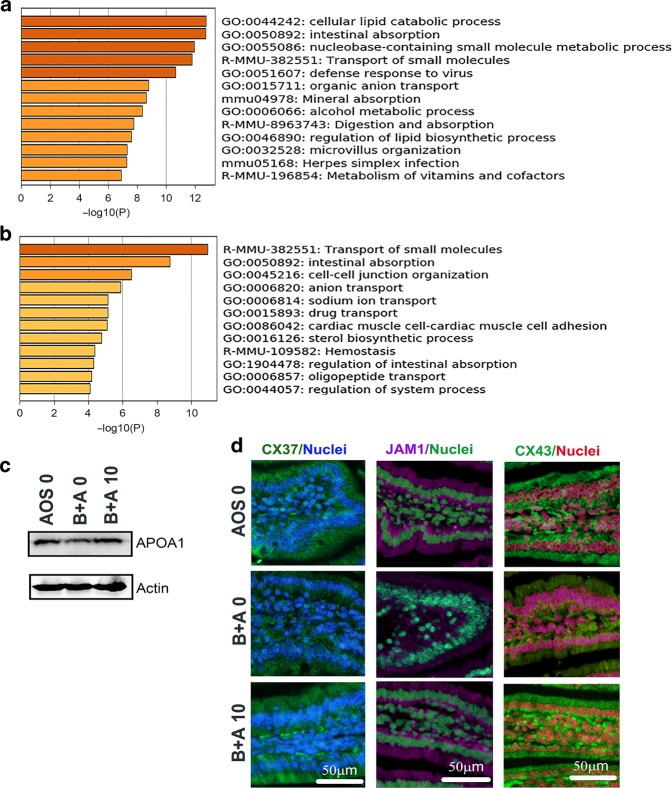
AOS improved enterocyte function. **a** Enrichment analysis for the cluster EMP only. **b** Enrichment analysis for the cluster EMD only. **c** WB of APOA1 in mouse small intestine samples. **d** IHF of Cx37, JAM1, and Cx43 in mouse small intestine samples in the three groups.

Moreover, the differentially expressed marker genes in EMD and EMP were further enriched separately (Fig. 5a, b). It was found that the functional pathway “microvillus organization” was enriched in EMP (Fig. 5a) and pathway “cell–cell junction organization” was enriched in the EMD cluster (Fig. 5b) which validated the data in the multiple enriched analysis. The enriched data for other groups are shown in Fig. S2. The data here suggested that busulfan disrupted microvilli and cell junctions in the small intestine and that AOS assisted in the reversal of this damage.

The data were further confirmed by IHF and WB. The level of the absorption protein APOA1 was reduced by busulfan while it was elevated by AOS (B + A 10) (Fig. 5c). In addition, the cell junction proteins JAM1, CX43, and Cx37 were reduced by busulfan while AOS (B + A 10) increased these protein levels (Fig. 5d). The data further confirmed that busulfan damaged the cell–cell junction by the decrease in the protein levels of junctional proteins. AOS could increase these protein levels to reconstruct the cell–cell junctions.

### AOS recovered the transcript factors by gene regulatory networks analysis

**Fig. 6:**
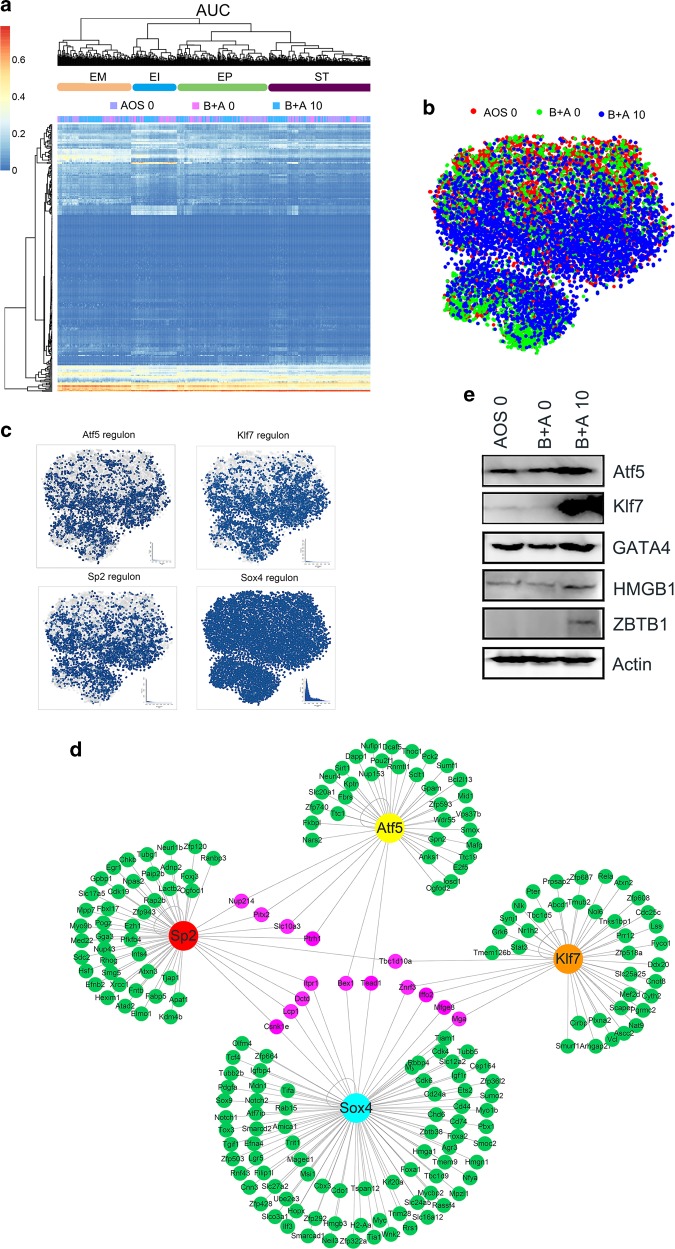
AOS recover the transcript factors by gene regulatory networks analysis. **a** SCENIC results on the mouse small intestine. Cluster labels are ST (stem, TA, TA-G1, TA-G2), EP (EP, EPE, EPL), EI (EIP, EID), EM (EMP, EMD). Different samples AOS 0, B + A 0, B + A 10 regulated different transcriptional factors. **b** Binary Regulon Activity colored by cell Proportions of different group. **c** Examples of reciprocal activity of four regulons Atf5, Klf7, Sp2, SOX4 on mouse small intestine single-cell data. Cells are colored according to the corresponding binary regulon activity. The inset illustrates the AUCell score distribution for the regulon. **d** Atf5, Klf7, SP2, SOX4 regulons and genes networks. Genes in green for individual regulon, genes in pink for multiple regulons. **e** WB analysis of regulons in mouse small intestine samples. (*n* > 6/group).

Small intestine cell identity and cell fate are governed by transcription factors and associated cofactors that work together to regulate the expression of target genes. The single-cell regulatory network inference and clustering (SCENIC) computational pipeline is an excellent tool to map gene regulatory network (GRN).^31^ In order to calculate the activity of every regulon in single-cell transcriptomes, the SCENIC AUCell algorithm was performed for cells in clusters ST (stem, TA, TA-G1, and TA-G2 cells), EP (EP, EPE, EPL), EI (EIP, EID), and EM (EMP, EMD).^31^ In our analysis, 184 regulons were identified, differentially expressed, and active in the three treatment groups of small intestine samples (Fig. 6a, Data File S2, S3). These active regulons were sample specific or cell type specific. Based on cell types, the active regulons could be divided into four major groups that regulons in ST, EP, EI, and EM (Fig. 6a). Some regulons were sample specific in AOS 0, B + A 0, or B + A 10 (Fig. 6a–c). Some of the important regulons were highly expressed in the B + A 10 group, such as Atf5, Klf7, and Sp2. While some regulons were commonly expressed in the AOS 0, B + A 0, and B + A 10 groups, such as the putative transcription factors SOX4, SOX9, GATA4, ELF1, and others (Fig. 6c, d). The data here suggested that busulfan disturbed the expression of transcription factors to further upset the gene expression in the intestinal cells. However, AOS can recover the expression of these transcription factors to improve the gene expression in these cells.

The protein levels of some regulons were explored by WB (Fig. 6e). We found that some transcription factors such as GATA4 and HMGB1 were decreased by busulfan, while they were increased by AOS (B + A 10). KLF7, ATF5, SOX4, and ZBTB1 were expressed in the B + A 10 group while there was less expression in other groups (Fig. 6e). The WB data further indicated that busulfan disrupted the transcription factors to upset gene expression in small intestinal cells. AOS produced the beneficial effects for the transcription factors. The data here suggested that AOS can regulate transcription factors to control gene expression which might be the reason why AOS can improve gene expression in intestinal cells.

### siRNA of mannose receptor (MR) blocked AOS effects on intestinal cells in vitro

**Fig. 7:**
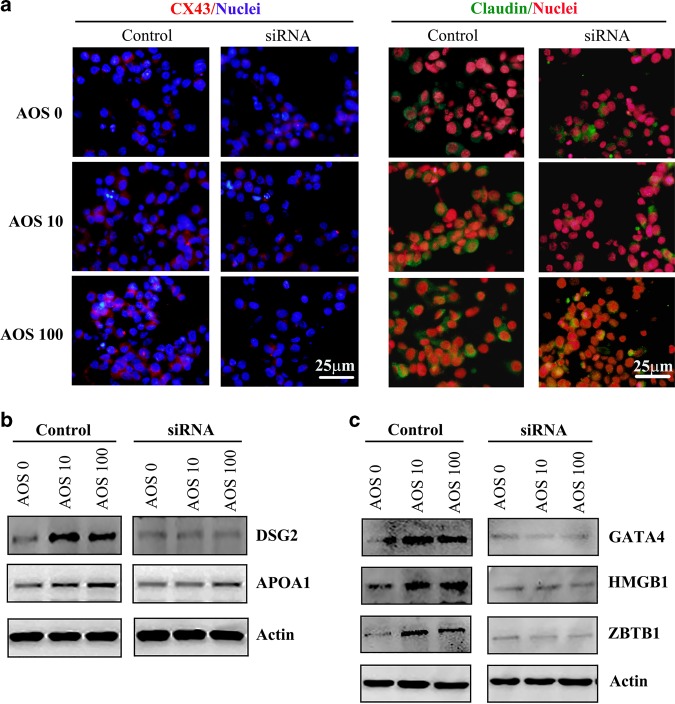
siRNA of mannose receptor blocked AOS effects on intestinal cells in vitro. **a** IHF of Cx437, claudin in IPEC-J2 cells the six groups. **b** WB of DSG2 and APOA1 in IPEC-J2 cells the six groups. **c** WB of GATA4, HMBG1, and ZBTB1 in IPEC-J2 cells the six groups. (>3 replications).

AOS are the degradation products of alginate (one type of marine polysaccharide from brown seaweed) which are composed of α-L-guluronate (G) and β-D-mannuronate (M) joined by 1, 4-glycoside bonds. The MR is an endocytic receptor containing eight C-type lectinlike domains (CTLDs) which can bind to glycoconjugates terminated in mannose, fucose, or glucose. We hypothesized that the function of AOS may be through MR.^32,33^ Then siRNAs of MR were used in this study in vitro with IPEC-J2 cells (swine intestinal cell line) to explore the mechanism of AOS effects on the recovering of mouse small intestinal. It was very interesting that AOS 10, and 100 μg/ml significantly increased the protein levels of CX43, claudin, DSG2, and APOA1 in IPEC-J2 cells which was constant with the in vivo data in mouse, however, AOS 10, and 100 μg/ml cannot increase the levels of these three proteins in the present of MR siRNA (Fig. 7a, b). We further determined the protein levels of the transcriptional factors GATA4, HMBG1 ad ZBTB1 in IPEC-J2 cells after MR siRNA treatment. We found that AOS 10, 100 μg/ml significantly elevated the expression of these three transcriptional factors which was constant with the in vivo data in mouse, however, AOS 10, and 100 μg/ml did not elevate the expression of these three proteins under MR siRNA treatment (Fig. 7c). The data suggested that AOS acts its function through MR signaling pathway.

### Data of plasma metabolome confirmed the sequencing data

**Fig. 8:**
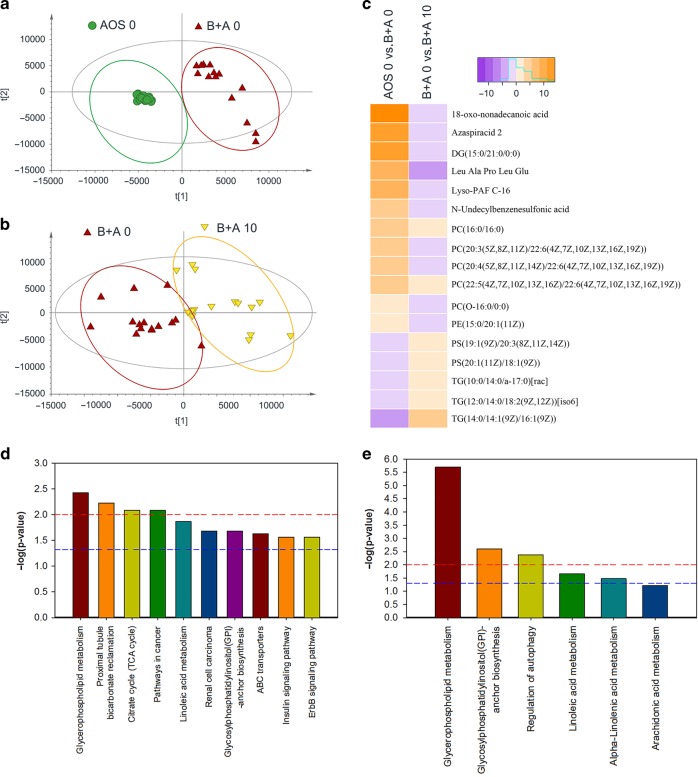
AOS improve plasma metabolome. **a** OPLS-DA of mouse plasma metabolites in AOS 0 and B + A 0 groups. **b** OPLS-DA of mouse plasma metabolites in B + A 0 and B + A 10 groups. **c** Change in metabolites under different treatments. **d** Enriched pathways of changed metabolites in AOS 0 vs. B + A 0. **e** Enriched pathways of changed metabolites in B + A 0 vs. B + A 10. (*n* > 13/group).

Since AOS improved the function of small intestine cells which are important for the absorption of nutrients, next we investigated whether AOS was beneficial to the blood metabolome (Data File S4). We found that busulfan changed plasma metabolites compared to the control (AOS 0; Fig. 8a), while AOS (B + A 10) altered plasma metabolites compared to busulfan alone (Fig. 8b). Interestingly, we found that AOS could reverse the effects of busulfan on metabolites. Our findings showed that busulfan increased some of the metabolites while they were decreased by AOS (B + A 10) (Fig. 8c); alternately, some metabolites were decreased by busulfan while they were increased by AOS (B + A 10; Fig. 8c). The altered metabolites were enriched and their function was determined by MetaboAnalyst. We found that 10 pathways were significantly enriched in the comparation of AOS 0 vs. B + A 0 (Fig. 8d). The most enriched pathway was “glycerophospholipid metabolism” which indicated that busulfan affected lipid metabolism in murine blood. Six pathways were enriched in the comparison of B + A 0 vs. B + A 10 (Fig. 8e). The most enriched pathway was also “glycerophospholipid metabolism” which indicated that the metabolism of glycerophospholipid was affected by busulfan while it could be rescued by AOS (B + A 10). The data in this section suggested that busulfan disturbed the systemic metabolites, while AOS recovered these metabolites. These findings confirmed the scRNA-seq analysis data, that AOS improved gene expression related to lipid metabolism which benefit the blood metabolites. The data here confirmed that AOS improved systemic metabolism which might be due to the enhancement in intestinal cell function.

## Discussion

The small intestine not only secretes mucus and hormones as a physical and chemical barrier to protect the body from pathogenic microbes but also digests and absorbs most of the nutrients into the blood.^6,28^ There are different cell types in the small intestine that work together to accomplish a wide array of tasks.^6,28^ It has been reported that chemotherapeutic drugs such as busulfan cause mucositis in cancer patients as they can damage the proliferation of cells in the small intestine. In the current investigation, we found that busulfan damaged small intestinal cells by injuring ER or mitochondria, disrupting the microvilli, and damaging cell membranes, especially cell junctions. AOS rescued the damage caused by busulfan leading to a recovery of ER or mitochondria, cure of the microvilli, and an increase in cell junction protein expression and cell junction formation. We explored the underlying mechanisms by investigating murine small intestines at a single-cell level using scRNA-seq analysis. As reported in a recently article,^28^ we found the same cell types in the small intestines of the mice, and the enterocytes developed under a pseudotime course.^6,28^ AOS improved small intestine function not only by recovering the proportion of different types of small intestinal cells, but also the gene and protein expression of these cell types. For differentially expressed genes in each cell type, enrichment analysis showed that the major functional pathways for each cell type were well correlated with their natural function. Furthermore, it was interesting to note that the enriched functional pathways for tuft cell was “T cell activation”; for EEC was “hormone secretion”; for Goblet and Paneth cells (GP) was “antibacterial humoral response”; for EMP and EMD included metabolic processes, absorption, regulation of microvilli length, microvillus organization, and cell–cell junction organization. The data suggested that the improved immune cells helped the recovery of enterocyte function. The most interesting findings of this study were that the scRNA-seq data and the ultrastructural changes, as determined by TEM, were well correlated. We found that AOS also benefited the expression of many transcriptional factors which further explains how AOS recovered gene and protein expression in the small intestine.^28^

AOS are composed of α-L-guluronate (G) and β-D-mannuronate (M) joined by 1, 4-glycoside bonds. The MR is a highly effective endocytic receptor and it has a broad binding specificity encompassing ligands. MR has been implicated in homeostatic processes, pathogen recognition, innate immune activation, cytokine production (both pro-inflammatory and anti-inflammatory). Moreover, MR can interact with other canonical pattern recognition receptors to mediate intracellular signaling.^32–34^ We found that inhibition of MR by its specific siRNA can block AOS function. The data suggested that AOS might act its function through MR signaling pathway.

The small intestine is the main organ for nutrient digestion and absorption into the circulatory system.^6,11,28^ We also explored plasma metabolome, and found that many metabolites were changed by busulfan compared to the control group; this confirmed that busulfan altered the mucosal barrier. AOS rescued the metabolites by reversing the concentration of plasma metabolites. Glycerophospholipid metabolism was the most enriched functional pathway for these changed metabolites. Glycerophospholipids, the structural components of biological membranes and the storage biologically active substances and molecules, play vital physiologic roles in cell growth, differentiation, migration, signal transduction, and apoptosis.^35^ Blood metabolic glycerophospholipids profile suggests the health stage of the GI tract. In current investigation, we found busulfan changed the blood glycerophospholipid metabolic profile which was reversed by AOS. Moreover, AOS rescued the busulfan disrupted small intestinal cells which are responsible for nutrition metabolism digestion and absorption. Furthermore, the recovering function in small intestine by AOS may through MR signaling pathway which is also involved in homeostatic processes. MR signaling pathway may also play some roles in the AOS recovering glycerophospholipid metabolism. The data confirmed that small intestinal cell functions have been recovered by AOS.

In summary, AOS improved small intestinal tuft cells, goblet cells and Paneth cells to help of the recovery of enterocytes then to enhance small intestine function which was confirmed by blood metabolome data. Our data proposes AOS as a novel, natural therapeutic drug for preventing small intestinal mucositis caused by anticancer drugs or other factors.

## Materials and methods [detailed Methods in supplemental information]

### Study design: (A) Mice exposure to busulfan and/or AOS

All animal procedures were approved and conducted in accordance with the Animal Institute of the Chinese Academy of Agricultural Sciences Animal Care and Use Committee. Mice were maintained under a light: dark cycle of 12:12 h and at a temperature of 23 ^o^C and humidity of 50–70%; they had free access to food (chow diet) and water.^36^

In our preliminary study (based on early report: Wan et al.^22^), AOS 10 mg/kg body weight dosing for two weeks was the best treatment condition for improving busulfan disrupted small intestine (1, 5, 10, 20 mg/kg body weight of AOS dosing for 1, 2, 3, and 4 weeks). Three-week-old ICR male mice were given a single injection of 40 mg/kg body weight (BW) of busulfan.^37^ The following day, the mice were dosed with ddH_2_O as the control or AOS 10 mg/kg BW via oral gavage (0.1 ml/mouse/day). Our preliminary experiments had found that 10 mg/kg was the best concentration for recovering the intestinal epithelial cell damage caused by busulfan. AOS dosing solution was freshly prepared on a daily basis in ddH_2_O. There were four treatment groups (30 mice/treatment): (1) vehicle control (ddH_2_O) designed as “AOS 0” group; (2) AOS 10 mg/kg BW designed as “AOS 10” group; (3) Busulfan alone (dosing with ddH_2_O) designated the “B + A 0” group; (4) Busulfan plus AOS 10 mg/kg BW designated as the “B + A 10” group. Gavage dosing took place every morning for two weeks. After treatment, the mice were humanely terminated for the collection of samples for different analyses.

### (B) IPEC-J2 cells exposure to MR siRNA and/or AOS

The porcine jejunal intestinal epithelial cell line IPEC-J2 cells were cultured in 1:1 Dulbecco’s Modified Eagle Medium (DMEM)/Ham’s F-12 mixture (Invitrogen Life Technologies, Carlsbad, CA, USA), supplemented with 10% (v/v) FBS (Invitrogen Life Technologies, Carlsbad, CA, USA), 1% (v/v) insulin-transferrin-selenite mixture (Gibco, Life Technologies, Paisley, Scotland, UK), 1% (v/v) penicillin–streptomycin (Gibco), further called cell medium.^38^ Cells were maintained at 37 °C in ambient atmosphere with 5% CO2.

The siRNAs (three sites) of MR and negative control (NC) were purchased from GenePharma (Shanghai, China) and used for the transfection of cells following the manufacturer’s instruction. The sequences for the siRNA and NC are provided in Table 1. Cells were cultured in 6-cm dishes and separated into six groups: NC + AOS 0, NC + AOS 10, NC + AOS 100, siRNA + AOS 0, siRNA + AOS 10, siRNA + AOS 100. After overnight attachment, the cells were first treated with NC or siRNAs (three sites together) for 8 h, then AOS was added into medium. The amount of NC added was 0.025 pmol/well, and the amount of siRNA was 0.025 pmol/well. Cells were collected for analysis at 48 h after transfection (40 h after AOS treatment).^39^

**Table 1:** Sequences for negative controls (NC) and mannose receptor 1(MR1) siRNAs.

	Sequencing
NC	
Sense	5′-UUCUCCGAACGUGUCACGUTT-3′
Anti-sense	5′-ACGUGACACGUUCGGAGAATT-3′
MR1-746(site 1)	
Sense	5′-GCGAGAGAUUAUGGAACAATT-3′
Anti-sense	5′-UUGUUCCAUAAUCUCUCGCTT-3′
MR1-2010(site 2)	
Sense	5′-GCCCACAACAACUCCUGAATT-3′
Anti-sense	5′-UUCAGGAGUUGUUGUGGGCTT-3′
MR1-4010(site 3)	
Sense	5′-GGAAGUGGCUUUGGUUGAATT-3′
Anti-sense	5′-UUCAACCAAAGCCACUUCCTT-3′

### Ultrastructural analysis of small intestine tissues by transmission electron microscopy (TEM)

The procedure for TEM analysis has been reported in our early article.^36^ Fifty nanometer sections were cut on a Leica Ultracut E equipped with a diamond knife (Diatome, Hatfield, PA). The sections were stained with uranyl acetate and viewed on a JEM-2010F TEM (JEOL Ltd., Japan).

### Single-cell library preparation, sequencing, and data analysis

#### Single-cell library preparation and sequencing

Single-cell libraries were constructed with 10x Genomics Chromium Single Cell 3′ Library & Gel Bead Kit v2 (10 × Genomics Inc., Pleasanton, CA, USA, 120237) following the manufacturer’s instructions. The single-cell samples collection was followed the reported procedures by Haber et al.^28^

#### Single sample analysis and aggregation

CellRanger v2.2.0 software (https://www.10xgenomics.com/) was applied to process the datasets with ‘--force-cells = 5000’ argument The 10x Genomics pre-built mouse genome for mm10-3.0.0 (https://support.10xgenomics.com/single-cell-gene-expression/software/downloads/latest) was referenced. After the CellRanger analysis, the gene-barcode matrices were processed with Seurat single-cell RNA seq analysis R package in Rstudio (v3.0).^40^

#### Subclustering, Gene Ontology enrichment analysis

After characterization of all cell clusters in mouse small intestine samples, cells were further clustered into different clusters based on their cell identity.

### Single-cell pseudo-time trajectory analysis

Monocle 2 (v2.8.0) was applied to determine the single-cell pseudo-time trajectory (http://cole-trapnell-lab.github.io/monocle-release/tutorials/).^30,41^ Monocle object was formed by Monocle implemented newCellDataSet function from Seurat object with lowerDetectionLimit = 0.5. The variable genes for ordering were got by Seurat.

### Single-cell regulatory network analysis

To find the gene regulatory networks during small intestine cell development, we performed regulatory network inference and clustering using SCENIC, a modified method for inferring gene regulatory networks from single-cell RNA seq data.^31^

### Plasma Metabolite measurements by LC-MS/MS

Plasma samples were collected and stored at −80 °C immediately. Before LC-MS/MS analysis, the samples were thawed on ice and processed to remove proteins. Then the samples detected by ACQUITY UPLC and AB Sciex Triple TOF 5600 (LC/MS) as reported in early article.^42,43^

### Histopathology analysis

Small intestinal tissues were fixed in 10% neutral formalin, paraffin embedded, cut into 5 μm sections, and stained with hematoxylin and eosin (H&E) for histopathological analysis.

### Immunofluorescent staining (IHF)

The procedure for IHF staining was reported in our recent publications.^36,44^ Table S1 lists the primary antibodies.

### Western blotting

Western blotting analysis was followed the procedure reported in our previous publications.^36,44^ Information for primary antibodies is in Table S1. Secondary donkey anti-goat Ab (Cat no.: A0181) was purchased from Beyotime Institute of Biotechnology (Shanghai, P.R. China), and goat anti-rabbit (Cat no.: A24531) Abs were bought from Novex® by Life Technologies (USA).

### Statistical analysis

The data were determined by SPSS statistical software (IBM Co., NY) with one-way analysis of variance (ANOVA) following by LSD multiple comparison test. All groups were compared with each other for every parameter. The data were shown as the mean ± SEM. Statistically significant was based on *p* < 0.05.

## Supplementary information

Supplementary Table S1

Supplementary Figure S1

Supplementary Figure S2

Data File S1, S2

Data File S3, S4

Supplemental information

## Data Availability

The 10x sequencing raw data are deposited in NCBI’s Gene Expression Omnibus under accession number: GSE131630.
