# Supplementary material for: Single-cell RNA sequencing analysis reveals alginate oligosaccharides preventing chemotherapy-induced mucositis
Source: Mucosal Immunol. 2020 Jan 3;13(3):437–48. doi: 10.1038/s41385-019-0248-z (PMC7181395; doi:10.1038/s41385-019-0248-z)
Supplement: Supplementary file 1 — Supplementary Table S1 [file 41385_2019_248_MOESM1_ESM.docx]

Supplementary Table 1. Primary antibody information

| **Gene symbol** | **Name** | **Cat. #** | **Predicted size** | **Source (Animal)** | **Company** |
| --- | --- | --- | --- | --- | --- |
| Vil1 | Villin | ab130751 | 93kDa | Rabbit | Abcam |
| DSG2 | Desmoglein 2 | ab150372 | 122kDa | Rabbit | Abcam |
| Bcl-2 | B cell lymphoma 2 | bs-4563R | 26kDa | Rabbit (polyclonal) | Beijing Biosynthesis Biotechnology CO. |
| p-PTEN | Phospho-PTEN | bs-3351R | 44kDa | Rabbit (polyclonal) | Beijing Biosynthesis Biotechnology CO. |
| Caspase 8 | Cysteine protease 8 | bs-0052R | 12/55kDa | Rabbit (polyclonal) | Beijing Biosynthesis Biotechnology CO. |
| Cx37 | Connexin37 | bs-4067R | 37kDa | Rabbit (polyclonal) | Beijing Biosynthesis Biotechnology CO. |
| Cx43 | Connexin 43 | bs-0651R | 42kDa | Rabbit (polyclonal) | Beijing Biosynthesis Biotechnology CO. |
| JAM1 | Junction adhesion molecule 1 | bs-3651R | 30kDa | Rabbit (polyclonal) | Beijing Biosynthesis Biotechnology CO. |
| APOA1 | Apolipoprotein A1 | bs-0849R | 28kDa | Rabbit (polyclonal) | Beijing Biosynthesis Biotechnology CO. |
| Atf5 | Activating transcription factor 5 | bs-12542R | 31kDa | Rabbit (polyclonal) | Beijing Biosynthesis Biotechnology CO. |
| Klf7 | Krueppel like factor 7 | bs-11865R | 33kDa | Rabbit (polyclonal) | Beijing Biosynthesis Biotechnology CO. |
| GATA4 | GATA binding factor 4 | bs-23982R | 49kDa | Rabbit (polyclonal) | Beijing Biosynthesis Biotechnology CO. |
| HMGB1 | High mobility group 1 | bs-0664R | 25kDa | Rabbit (polyclonal) | Beijing Biosynthesis Biotechnology CO. |
| ZBTB1 | Zinc finger and BTB domain containing 1 | bs-13563R | 82kDa | Rabbit (polyclonal) | Beijing Biosynthesis Biotechnology CO. |
| actin | actin | Ab3280 | 42kDa | Rabbit (polyclonal) | Abcam |
