# Supplementary figures and images for: Single-cell RNA sequencing analysis reveals alginate oligosaccharides preventing chemotherapy-induced mucositis
Source: Mucosal Immunol. 2020 Jan 3;13(3):437–48. doi: 10.1038/s41385-019-0248-z (PMC7181395; doi:10.1038/s41385-019-0248-z)

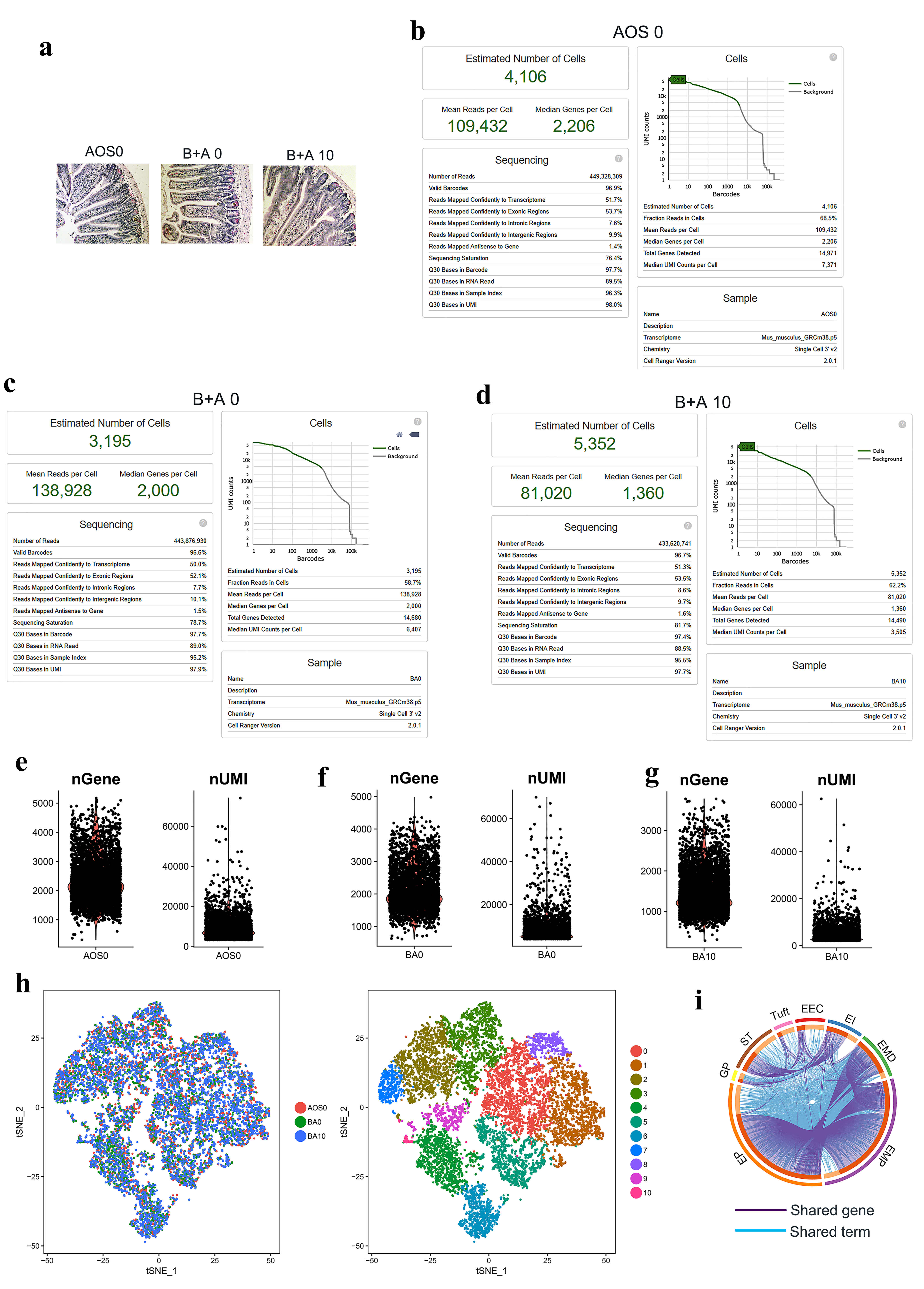

Supplement: Supplementary file 2 — Supplementary Figure S1 [file 41385_2019_248_MOESM2_ESM.tif]
