# Supplementary material for: Single-cell RNA sequencing analysis reveals alginate oligosaccharides preventing chemotherapy-induced mucositis
Source: Mucosal Immunol. 2020 Jan 3;13(3):437–48. doi: 10.1038/s41385-019-0248-z (PMC7181395; doi:10.1038/s41385-019-0248-z)
Supplement: Supplementary file 3 — Supplementary Figure S2 [file 41385_2019_248_MOESM3_ESM.pdf]

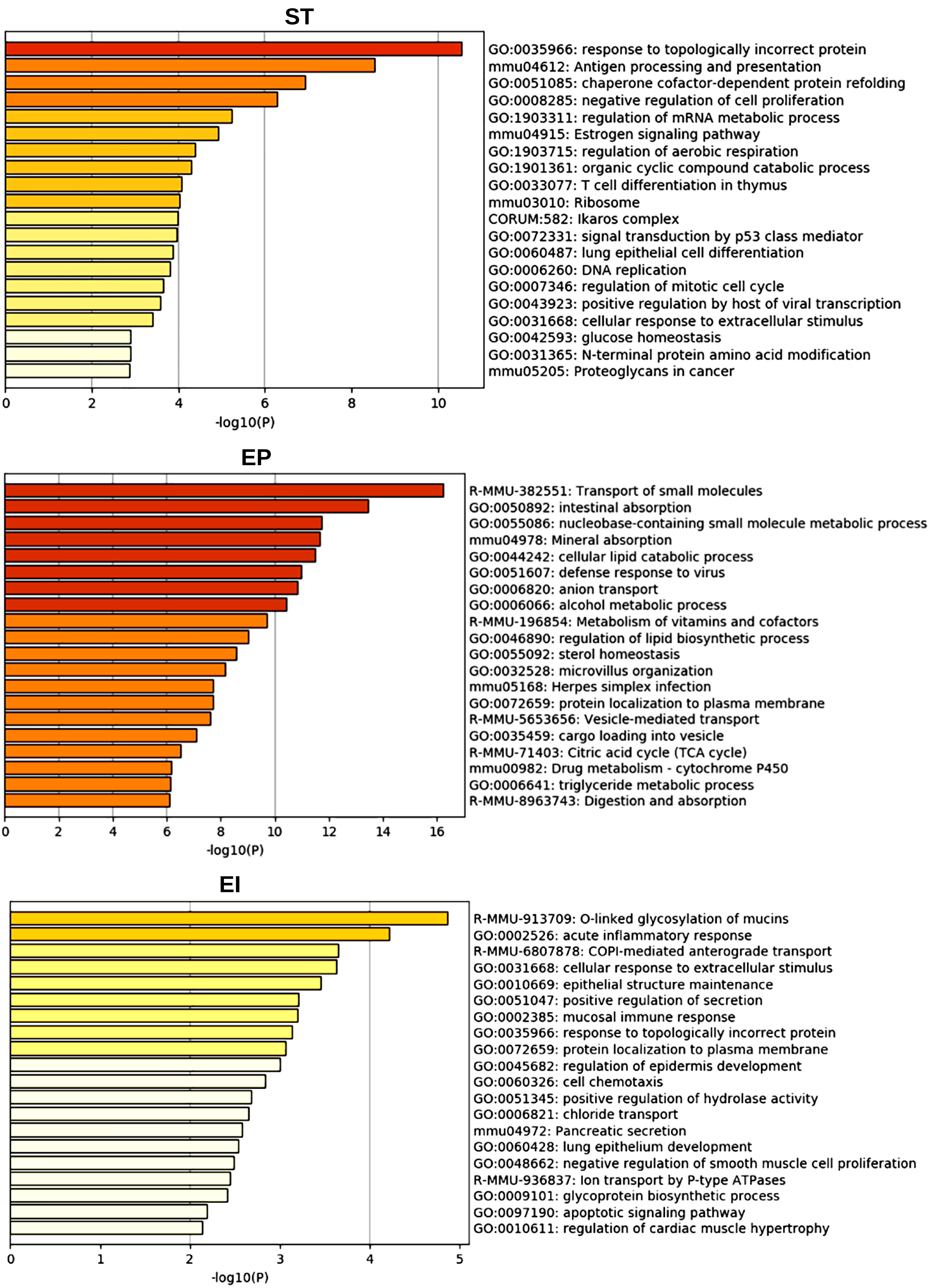
ST
GO:0035966: response to topologically incorrect protein
mmu04612: Antigen processing and presentation
GO:0051085: chaperone cofactor-dependent protein refolding
GO:0008285: negative regulation of cell proliferation
GO:1903311: regulation of mRNA metabolic process
mmu04915: Estrogen signaling pathway
GO:1903715: regulation of aerobic respiration
GO:1901361: organic cyclic compound catabolic process
GO:0033077: T cell differentiation in thymus
mmu03010: Ribosome
CORUM:582: Ikaros complex
GO:0072331: signal transduction by p53 class mediator
GO:0060487: lung epithelial cell differentiation
GO:0006260: DNA replication
GO:0007346: regulation of mitotic cell cycle
GO:0043923: positive regulation by host of viral transcription
GO:0031668: cellular response to extracellular stimulus
GO:0042593: glucose homeostasis
GO:0031365: N-terminal protein amino acid modification
mmu05205: Proteoglycans in cancer
0
2
4
6
8
10
-log10(P)
EP
R-MMU-382551: Transport of small molecules
GO:0050892: intestinal absorption
GO:0055086: nucleobase-containing small molecule metabolic process
mmu04978: Mineral absorption
GO:0044242: cellular lipid catabolic process
GO:0051607: defense response to virus
GO:0006820: anion transport
GO:0006066: alcohol metabolic process
R-MMU-196854: Metabolism of vitamins and cofactors
GO:0046890: regulation of lipid biosynthetic process
GO:0055092: sterol homeostasis
GO:0032528: microvillus organization
mmu05168: Herpes simplex infection
GO:0072659: protein localization to plasma membrane
R-MMU-5653656: Vesicle-mediated transport
GO:0035459: cargo loading into vesicle
R-MMU-71403: Citric acid cycle (TCA cycle)
mmu00982: Drug metabolism - cytochrome P450
GO:0006641: triglyceride metabolic process
R-MMU-8963743: Digestion and absorption
0
2
4
6
8
10
12
14
16
-log10(P)
EI
R-MMU-913709: O-linked glycosylation of mucins
GO:0002526: acute inflammatory response
R-MMU-6807878: COPI-mediated anterograde transport
GO:0031668: cellular response to extracellular stimulus
GO:0010669: epithelial structure maintenance
GO:0051047: positive regulation of secretion
GO:0002385: mucosal immune response
GO:0035966: response to topologically incorrect protein
GO:0072659: protein localization to plasma membrane
GO:0045682: regulation of epidermis development
GO:0060326: cell chemotaxis
GO:0051345: positive regulation of hydrolase activity
GO:0006821: chloride transport
mmu04972: Pancreatic secretion
GO:0060428: lung epithelium development
GO:0048662: negative regulation of smooth muscle cell proliferation
R-MMU-936837: Ion transport by P-type ATPases
GO:0009101: glycoprotein biosynthetic process
GO:0097190: apoptotic signaling pathway
GO:0010611: regulation of cardiac muscle hypertrophy
0
1
2
3
4
5
-log10(P)

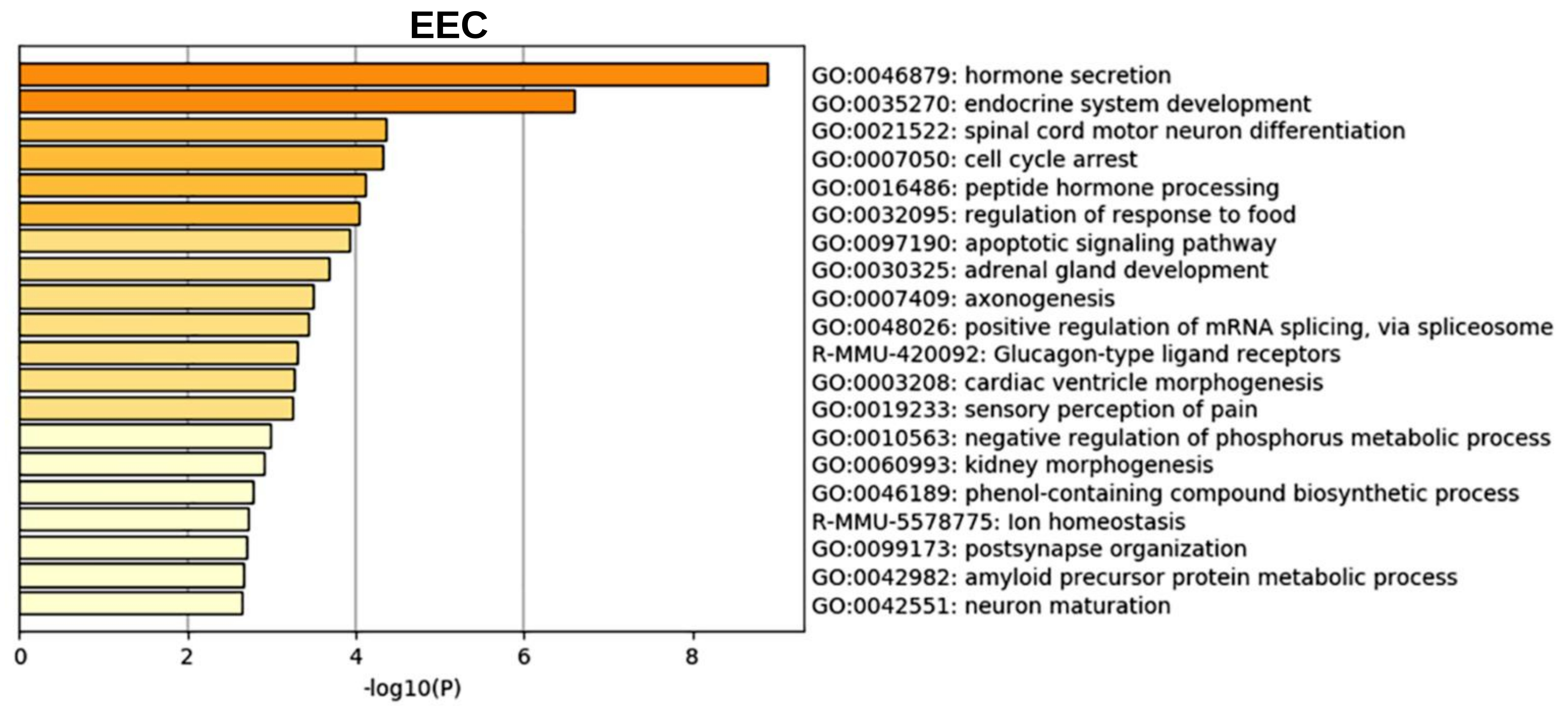
EEC
GO:0046879: hormone secretion
GO:0035270: endocrine system development
GO:0021522: spinal cord motor neuron differentiation
GO:0007050: cell cycle arrest
GO:0016486: peptide hormone processing
GO:0032095: regulation of response to food
GO:0097190: apoptotic signaling pathway
GO:0030325: adrenal gland development
GO:0007409: axonogenesis
GO:0048026: positive regulation of mRNA splicing, via spliceosome
R-MMU-420092: Glucagon-type ligand receptors
GO:0003208: cardiac ventricle morphogenesis
GO:0019233: sensory perception of pain
GO:0010563: negative regulation of phosphorus metabolic process
GO:0060993: kidney morphogenesis
GO:0046189: phenol-containing compound biosynthetic process
R-MMU-5578775: Ion homeostasis
GO:0099173: postsynapse organization
GO:0042982: amyloid precursor protein metabolic process
GO:0042551: neuron maturation
0
2
4
6
8
-log10(P)

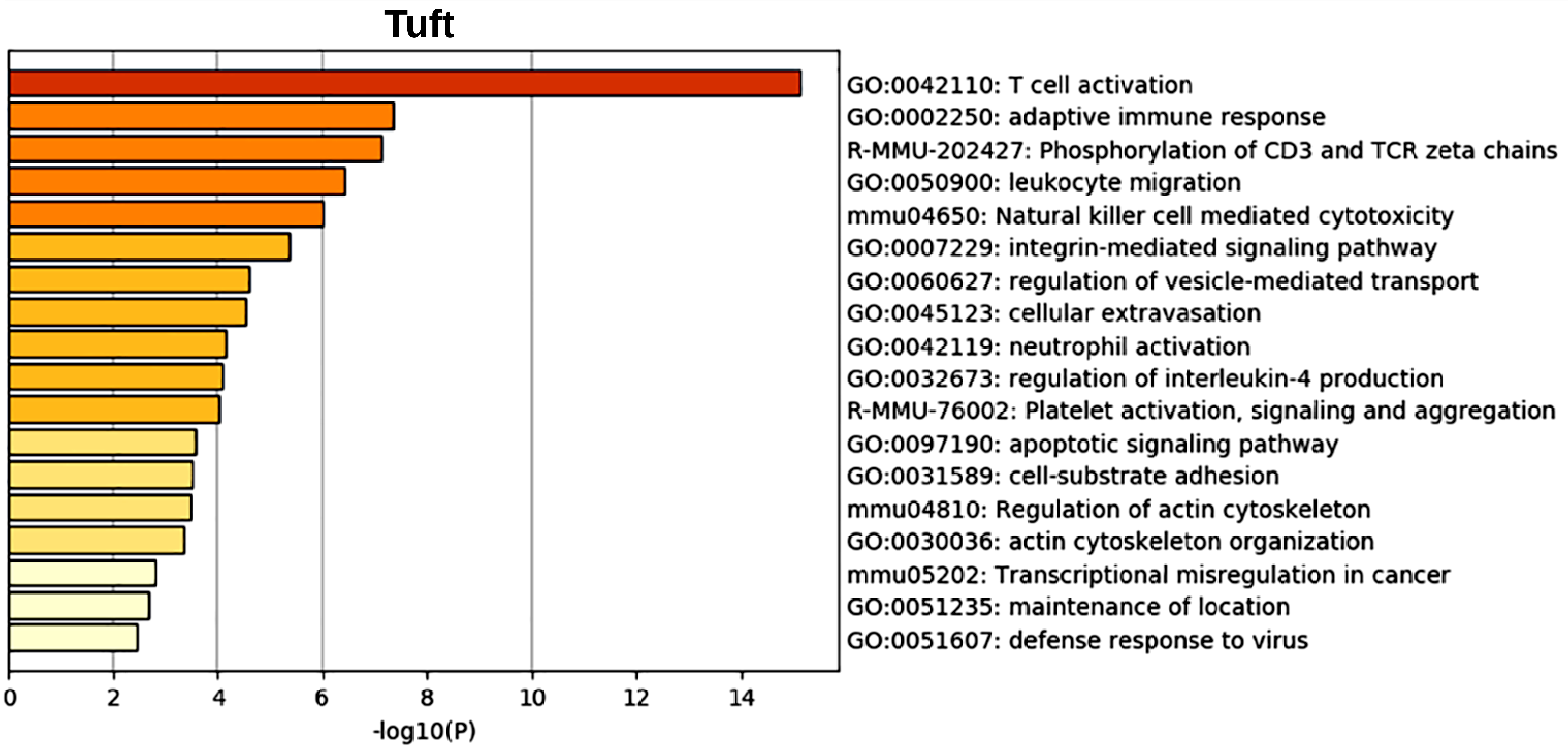
Tuft
GO:0042110: T cell activation
GO:0002250: adaptive immune response
R-MMU-202427: Phosphorylation of CD3 and TCR zeta chains
GO:0050900: leukocyte migration
mmu04650: Natural killer cell mediated cytotoxicity
GO:0007229: integrin-mediated signaling pathway
GO:0060627: regulation of vesicle-mediated transport
GO:0045123: cellular extravasation
GO:0042119: neutrophil activation
GO:0032673: regulation of interleukin-4 production
R-MMU-76002: Platelet activation, signaling and aggregation
GO:0097190: apoptotic signaling pathway
GO:0031589: cell-substrate adhesion
mmu04810: Regulation of actin cytoskeleton
GO:0030036: actin cytoskeleton organization
mmu05202: Transcriptional misregulation in cancer
GO:0051235: maintenance of location
GO:0051607: defense response to virus
0
2
4
6
8
10
12
14
-log10(P)

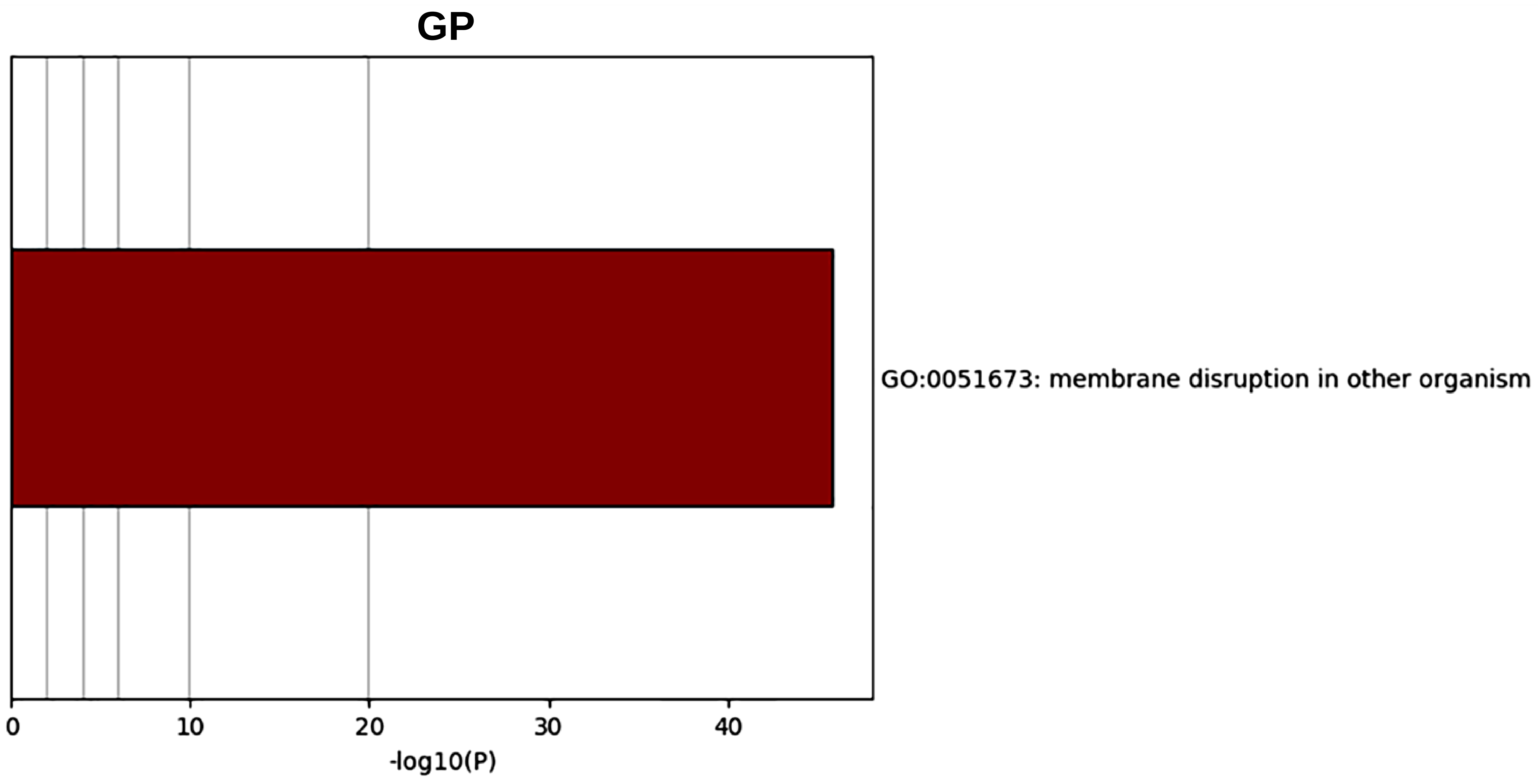
GP
GO:0051673: membrane disruption in other organism
0
10
20
30
40
-log10(P)
