# Supplementary material for: Single-cell RNA sequencing analysis reveals alginate oligosaccharides preventing chemotherapy-induced mucositis
Source: Mucosal Immunol. 2020 Jan 3;13(3):437–48. doi: 10.1038/s41385-019-0248-z (PMC7181395; doi:10.1038/s41385-019-0248-z)
Supplement: Supplementary file 6 — Supplemental information [file 41385_2019_248_MOESM6_ESM.docx]

**Supplemental information:**

**Detailed Methods**

***Ultrastructural analysis of small intestine tissues by Transmission Electron Microscopy (TEM)***

The procedure for TEM analysis has been reported in our early article.**^32^** Briefly, the freshly collected intestinal tissue was fixed in 2% glutaraldehyde in sodium phosphate buffer (pH 7.2) for 2 h. Samples were then washed 5 times in phosphate buffer (PBS) and fixed in 1% OsO4 for 1 h in the dark followed by several washes in PBS. Subsequently, the samples were dehydrated in ethanol using a series of increasing concentrations and infiltrated in Spur’s embedding medium in propylene epoxide with increased concentration. Afterwards, the specimens were polymerized for 12 h at 37 °C, 12 h at 45 °C, and 48 h at 60 °C in embedding medium. Fifty nanometer sections were cut on a Leica Ultracut E equipped with a diamond knife (Diatome, Hatfield, PA). The sections were stained with uranyl acetate and viewed on a JEM-2010F TEM (JEOL Ltd., Japan).

***Single cell library preparation, sequencing and data analysis***

***Single cell library preparation and sequencing.*** Single cell libraries were constructed with 10x Genomics Chromium Single Cell 3′ Library & Gel Bead Kit v2 (10×Genomics Inc., Pleasanton, CA, USA, 120237) following the manufacturer’s instructions. The single cell samples collection was followed the reported procedures by Haber et al.**^28^** Briefly, mouse small intestine was collected and Peyer’s patches were removed, then the small intestine tissue was washed with PBS. Then, the tissue was incubated in 20 mM EDTA-PBS for 90min on ice with shaking every 30 min. After 90min incubation, the samples were shaken very vigorously and the supernatant was collected into a new tube. Then the samples were incubated with fresh 20 mM EDTA-PBS for 30min on ice, and the supernatant was collected again. Four fractions were collected and combined together. The samples were centrifuged at 300g for 3 min, then the cell pellet was collected, and washed with PBS twice with same centrifugation program. The cells were digested by TrypLE express (Invitrogen) for 1 min at 37°C. The single cells were got by filtrated by a 40 μm filter. Then the cells were washed twice with PBS solution supplemented with 0.04% bovine serum albumin (BSA, Sigma, St. Louis, MO, USA, A1933). Trypan blue staining with a haemocytometer (Bio-Rad, Hercules, CA, USA, TC20) was applied to detect the cell viability. Six mouse intestine cells were collected individually, then the cells from the six samples were combined together. Then the concentration was 1000 cells/μl for loading to the single cell chip (one/group). Chromium 10x Single Cell System (10×Genomics, Pleasanton, CA, USA) was applied to form the Gel-Bead in Emulsions (GEMs) system. Then cells were barcoded and cDNA library was constructed. The sequencing was done by an Illumina HiSeq X Ten sequencer (Illumina, San Diego, CA, USA) with pair end 150 bp (PE150) reads.

***Single sample analysis and aggregation.*** CellRanger v2.2.0 software (https://www.10xgenomics.com/) was applied to process the datasets with ‘--force-cells = 5000’ argument The 10x Genomics pre-built mouse genome for mm10-3.0.0 ([https: //support.10xgenomics.com/single-cell-gene-expression/software/downloads/latest](https://support.10xgenomics.com/single-cell-gene-expression/software/downloads/latest)) was referenced. After the CellRanger analysis, the gene-barcode matrices were processed with Seurat single cell RNA seq analysis R package in Rstudio (v3.0).**^34^** Cells with minimal genes less than 200 and genes expressed in less than 3 cells were removed to keep high-quality datasets for downstream analysis. After normalization, the three datasets (from three treatment groups) were then merged together with Seurat RunMultiCCA function. The characterized cell clusters were reviewed by Seurat RunTSNE function based on the t-distributed Stochastic Neighbor Embedding (tSNE) algorithm with default settings. The cell clusters were calculated with FindClusters function. Seurat FindAllMarkers function was applied to find cell cluster markers.

***Subclustering, Gene Ontology enrichment analysis*.** After characterization of all cell clusters in mouse small intestine samples, cells were further clustered into different clusters based on their cell identity. To obtain the same type of cells for downstream analysis, SubsetData function was applied. After clustering, cluster-specific markers genes were found out by the FindAllMarkers function. The marker genes were used for the enrichment analysis by Metascape (<http://metascape.org>).

***Single-cell pseudo-time trajectory analysis*.** Monocle 2 (v2.8.0) was applied to determine the single-cell pseudo-time trajectory (<http://cole-trapnell-lab.github.io/monocle-release/tutorials/>).**^30,35^** Monocle object was formed by Monocle implemented newCellDataSet function from Seurat object with lowerDetectionLimit = 0.5. The variable genes for ordering were got by Seurat. Dimensionality was constructed by DDRTree method with regression of the number of UMIs. The root state was collected following their Seurat cell identity information and branch-specific gene expression was calculated using Monocle implemented BEAM function. The branched heatmap was further constructed by “plot_genes_branched_heatmap” function.

***Single cell regulatory network analysis*.** To find the gene regulatory networks during small intestine cell development, we performed regulatory network inference and clustering using SCENIC (https://github.com/aertslab/SCENIC), a modified method for inferring gene regulatory networks from single cell RNA seq data.**^31^** In the single-cell RNA-seq expression matrix, each column represents a cell ID and each row represents a gene, was applied for the analysis. Then geneFiltering function was applied to remove genes with UMI counts across all samples less than 100 and expressed in less than 1 % of cells. Then GENIE3 was used to infer co-expression matrix containing potential regulators. To identify potential direct-binding targets, RcisTarget was applied based on DNA-motif analysis and we used databases (mm10) that score the motifs in the promoter of the genes (up to 500 bp upstream the TSS), and in the 10 kb around the TSS (+/-10 kb). AUCell algorithm was applied to calculate regulon activity in each cell and convert the network activity into ON/OFF (binary activity matrix) with default settings.

***Plasma Metabolite measurements by LC-MS/MS***

Plasma samples were collected and stored at -80 °C immediately. Before LC-MS/MS analysis, the samples were thawed on ice and processed to remove proteins. Then the samples detected by ACQUITY UPLC and AB Sciex Triple TOF 5600 (LC/MS) as reported in early article.**^36,37^**

The HPLC conditions employed an ACQUITY UPLC BEH C18 column (100 mm × 2.1 mm, 1.7 μm), solvent A (aqueous solution with 0.1% (v/v) formic acid) and solvent B (acetonitrile with 0.1% (v/v) formic acid) with a gradient program:

| **Time** | **A%** | **B%** |
| --- | --- | --- |
| 0 | 95 | 5 |
| 2 | 80 | 20 |
| 4 | 75 | 25 |
| 9 | 40 | 60 |
| 17 | 0 | 100 |
| 19 | 0 | 100 |
| 19.1 | 95 | 5 |
| 20.1 | 95 | 5 |

The flow rate was 0.4 mL/min and the injection volume was 5μL.

The mass spectrometry program with ESI was:

| **Parameters** | Positive ion | Positive ion |
| --- | --- | --- |
| Nebulizer Gas (GS1, PSI) | 40 | 40 |
| Auxiliary Gas (GS2, PSI) | 40 | 40 |
| Curtain Gas (CUR, PSI） | 35 | 35 |
| Ion Source Temperature （℃） | 550 | 550 |
| Ion Spray Voltage (V) | 5500 | 4500 |
| Declustering Potential (DP ,V) | 100 | -100 |
| Mass Scan Range (TOF MS scan) | 70-1000 | 70-1000 |
| Collision Energy （TOF MS scan, eV） | 10 | -10 |
| Mass Scan Range (Product Ion scan) | 50-1000 | 50-1000 |
| Collision Energy (Product Ion scan,eV） | 30 | 30 |
| Interface Heater Temperature (℃) | 550 | 600 |

Progenesis QI v2.3 (Nonlinear Dynamics, Newcastle, UK) was applied to normalize the peaks. Then the Human Metabolome Database (HMDB), Lipidmaps (v2.3) and METLIN software were applied to qualify the data. Moreover, the data were processed with SIMCA software (version 14.0, Umetrics, Umeå, Sweden) following by the pathway enrichment analysis by KEGG database ([http://www.genome.jp/KEGG/pathway.html](http://www.genome.jp/kegg/pathway.html)).

***Histopathology analysis***

Small intestinal tissues were fixed in 10% neutral formalin, paraffin embedded, cut into 5 μm sections, and stained with hematoxylin and eosin (H&E) for histopathological analysis.

***Immunofluorescent staining (IHF)***

The procedure for immunofluorescent staining was reported in our recent publications.**^32,38^** Table S1 lists the primary antibodies. Briefly, small intestine sections (5 μm) were prepared and subjected to antigen retrieval, then the sections were first blocked with normal goat serum in PBS, followed by incubation (1:150 in PBS-1% BSA) with primary antibodies at 4 ^o^C overnight. After a brief wash, sections were incubated with a goat anti-rabbit or donkey anti-goat secondary Abs (1:100 in PBS; Beyotime Institute of Biotechnology, Shanghai, P.R. China) at room temperature for 30 min and then counterstained with 4',6-diamidino-2-phenylindole (DAPI). The stained sections were visualized using a Nikon Eclipse TE2000-U fluorescence microscope (Nikon, Inc., Melville, NY), and the captured fluorescent images were analyzed using MetaMorph software.

***Western blotting***

Western blotting analysis was followed the procedure reported in our previous publications. **^32,38^** Briefly, small intesine tissue samples were lysed in RIPA buffer containing the protease inhibitor cocktail from Sangong Biotech, Ltd. (Shanghai, China). Protein concentration was determined by BCA kit (Beyotime Institute of Biotechnology, Shanghai, China). Information for primary antibodies is in Table S1. Secondary donkey anti-goat Ab (Cat no.: A0181) was purchased from Beyotime Institute of Biotechnology (Shanghai, P.R. China), and goat anti-rabbit (Cat no.: A24531) Abs were bought from Novex^®^ by Life Technologies (USA). Fifty micrograms of total protein per sample were loaded onto 10% SDS polyacrylamide electrophoresis gels. The gels were transferred to a polyvinylidene fluoride (PVDF) membrane at 300 mA for 2.5 h at 4 ℃. Then, the membranes were blocked with 5% BSA for 1 h at RT, followed by three washes with 0.1% Tween-20 in TBS (TBST). The membranes were incubated with primary Abs diluted with 1:500 in TBST with 1% BSA overnight at 4 ℃. After three washes with TBST, the blots were incubated with the HRP-labelled secondary goat anti-rabbit or donkey anti-goat Ab respectively for 1 h at RT. After three washes, the blots were imaged. The experiment was performed with 6 individual samples/group.

**Data availability:** The 10x sequencing raw data are deposited in NCBI’s Gene Expression Omnibus under accession number: GSE131630.

***Statistical analysis***

The data were determined by SPSS statistical software (IBM Co., NY) with one-way analysis of variance (ANOVA) following by LSD multiple comparison test. All groups were compared with each other for every parameter. The data were shown as the mean ± SEM. Statistically significant was based on p<0.05.
